# The Ethylene Biosynthesis Genes *ACS2* and *ACS6* Modulate Disease Severity of *Verticillium dahliae*

**DOI:** 10.3390/plants9070907

**Published:** 2020-07-17

**Authors:** Eirini G. Poulaki, Maria-Dimitra Tsolakidou, Danai Gkizi, Iakovos S. Pantelides, Sotirios E. Tjamos

**Affiliations:** 1 Laboratory of Phytopathology, Agricultural University of Athens, 75 Iera Odos, 11855 Athens, Greece; poulakie@gmail.com (E.G.P.); danai_gk@hotmail.com (D.G.); 2 Department of Agricultural Sciences, Biotechnology and Food Science, Cyprus University of Technology, 30 Arch. Kyprianos Str., Limassol 3036, Cyprus; maria.tsolakidou@cut.ac.cy (M.-D.T.); iakovos.pantelides@cut.ac.cy (I.S.P.)

**Keywords:** 1-aminocyclopropane-1-carboxylic acid synthase, 1-aminocyclopropane-1-carboxylic acid, *Arabidospsis thaliana*, ethylene, phytohormones

## Abstract

*Verticillium dahliae* is one of the most destructive soilborne plant pathogens since it has a broad host range and there is no chemical disease management. Therefore, there is a need to unravel the molecular interaction between the pathogen and the host plant. For this purpose, we examined the role of 1-aminocyclopropane-1-carboxylic acid synthases (ACSs) of *Arabidopsis thaliana* upon *V. dahliae* infection. We observed that the *acs2*, *acs6*, and *acs2/6* plants are partially resistant to *V. dahliae*, since the disease severity of the *acs* mutants was lower than the wild type (wt) Col-0 plants. Quantitative polymerase chain reaction analysis revealed that *acs2*, *acs6*, and *acs2/6* plants had lower endophytic levels of *V. dahliae* than the wt. Therefore, the observed reduction of the disease severity in the *acs* mutants is rather associated with resistance than tolerance. It was also shown that *ACS2* and *ACS6* were upregulated upon *V. dahliae* infection in the root and the above ground tissues of the wt plants. Furthermore, the addition of 1-aminocyclopropane-1-carboxylic acid (ACC) and aminooxyacetic acid (AOA), the competitive inhibitor of ACS, in wt *A. thaliana*, before or after *V. dahliae* inoculation, revealed that both substances decreased *Verticillium* wilt symptoms compared to controls irrespectively of the application time. Therefore, our results suggest that the mechanism underpinning the partial resistance of *acs2* and *acs6* seem to be ethylene depended rather than ACC related, since the application of ACC in the wt led to decreased disease severity compared to control.

## 1. Introduction

*Verticillium dahliae* Kleb. is one of the most destructive soil inhabiting fungal pathogens with a worldwide distribution, infecting a wide range of plants, including vegetables, fruits, flowers, oilseed crops, fiber crops, and woody perennials [1,2]. The disease management of *V. dahliae* is particularly difficult because the fungus survives in the soil as resting structures, microsclerotia, for several years [2], and there are no effective chemical treatments to control the disease. Therefore, it is essential to investigate the host plant–*V. dahliae* interaction in order to identify the key components that make this interaction disease compatible and develop resistant cultivars or chemical compounds interfering with the pathogenicity mechanisms.

Ethylene has been long implicated as a pathogenicity factor in the *V. dahliae* interaction with plants. The *Verticillium* symptoms of epinasty, stunting, premature senescence, and leaf abscission have been associated with ethylene (ET) [3,4]. Interestingly, the defective in ET perception Arabidopsis mutant *etr1* is partially resistant to *V. dahliae* [5]. It has been shown that *etr1* plants have lower *Verticillium* disease severity levels than wild type (wt). This observation was associated with reduced endophytic levels of *V. dahliae* DNA in the *etr1* plants compared to wt [5]. On the other hand, the ET insensitive mutants *ein2-1*, *ein4-1*, and *ein6-1* are more susceptible to *V. dahliae* than wt [5,6]. The ethylene response 1 (ETR1) and ethylene insensitive 4 (EIN4) are members of different subfamilies of ET receptors [7], while the ethylene insensitive 2 (EIN2), ethylene insensitive 3 (EIN3), and ethylene insensitive 5 (EIN5) genes are positive regulators of ET responses, acting downstream of ETR1, which acts as a negative regulator of ET; therefore, these differences may explain the different responses of *etr1* and *ein* mutants to *V. dahliae* [6,7].

In contrast to the extensively studied responses of ethylene perception mutants upon *V. dahliae* infection, the interaction of ethylene biosynthesis mutants with *Verticillum* has not been reported yet. The rate-limiting step of ET synthesis in plants is the conversion of S-adenosyl-methionine (S-AdoMet) to 1-aminocyclopropane-1-carboxylic acid (ACC) by ACC synthase (ACS); therefore, to this day, the study of ACS has attracted much scientific attention [8,9,10,11]. ACS is encoded by a multigene family in every plant species examined [10]. The Arabidopsis genome contains 12 genes annotated as ACS (ACS1–12), dispersed among the five chromosomes [10]. The expression of the *ACS* genes in Arabidopsis is highly regulated by a variety of endogenous or environmental signals [11]. High-order ACS mutants with reduced ET induction are more susceptible to *Pseudomonas syringae* pv *tomato* than wt, demonstrating a positive role of ET in plant bacterial resistance [12]. High levels of *ACS2* and *ACS6* expression were observed, approximately 185- and 33-fold, respectively, at 6 h post-inoculation, in response to *P. syringae* pv *tomato* inoculation in Arabidopsis. However, *ACS2* and *ACS6* did not contribute to *P. syringae* pv *tomato*-induced ET production. The lack of *ACS2*/*ACS6* contribution suggests that *P. syringae* pv *tomato* effector(s) might target these two ACS isoforms and impede their function either directly or indirectly [12]. Similarly, *ACS2* and *ACS6* are involved in *Botrytis cinerea*-induced ethylene production [13,14]. It is evident that *ACS2* and *ACS6* have a role in leaf invasion by necrotrophs. Therefore, it will be scientifically interesting to investigate their role in plant infection by a root invading fungus, like *V. dahliae*.

The main objectives of the study were to (i) screen the resistance of the *Arabidospsis thaliana* mutants *acs2*, *acs6*, *acs1*, and *acs2/6* against *V. dahliae*, (ii) investigate whether the observed resistance of *acs2*, *acs6*, and *acs2/6* is associated with reduced endophytic colonization by *V. dahliae* and vice versa for *acs1,* (iii) detect the expression of *ACS2* and *ACS6* in the root and above ground plant tissues of wild type (wt) Col-0 plants, upon pathogen invasion, and (iv) identify whether the observed resistance of *acs2* and *acs6* is due to low ACC or ethylene production.

## 2. Results

### 2.1. Verticillium Wilt Symptom Development

**Figure 1:**
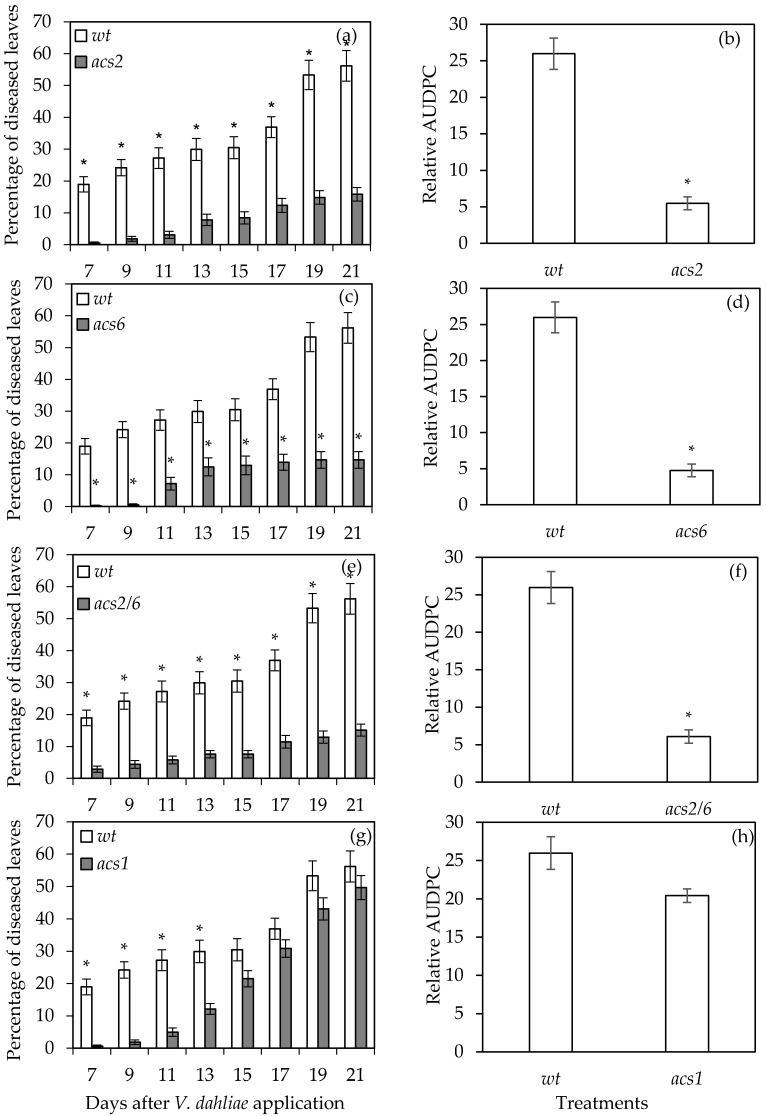
Disease severity (**a**,**c**,**e**,**g**) and relative area under the disease progress curve (AUDPC) (**b**,**d**,**f**,**h**) on *Arabidopsis thaliana* wild type and 1-aminocyclopropane-1-carboxylic acid synthase (*acs)2*, *acs6*, *acs2/6*, and *acs1* mutants inoculated with *Verticillium dahliae*. Each treatment consisted of 10 plants and the experiment was repeated three times. Columns represent means of the three replications (30 plants) and the vertical bars indicate standard errors. Columns with asterisks (*) are significantly different (*p* < 0.05) from wild type (wt) according to a *t*-test.

**Figure 2:**
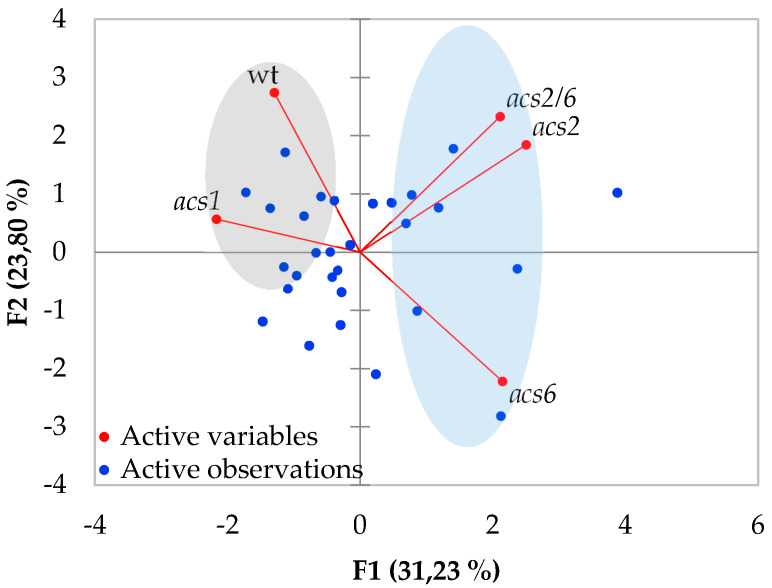
The principal component analysis of the relative AUDPC values of the *acs* and wt reveals clustered genotypes between the *acs2*, *acs2/6*, *acs6*, and wt, *acs1*. The percentage of variation explained by the plotted principal coordinates is indicated on the axes.

The inoculation of *acs2*, *acs6*, *acs2/6*, and *acs1* with *V. dahliae* revealed the partial resistance of *acs2*, *acs6*, and *acs2/6*. The first symptoms appeared in the form of wilting, especially on older leaves at seven days post-inoculation (dpi), and were recorded until 21 dpi (Figure 1). The *acs2*, *acs6*, and *acs2/6* displayed significantly less symptoms than wt throughout the experimental time period. At the final disease scoring day (21 dpi), the disease severity was ca. 15% in *acs2*, *acs6*, and *acs2/6*, while in the wt it was 56%. Consequently, statistical analysis on the relative area under the disease progress curve (AUDPC) values showed that disease severity in *acs2*, *acs6*, and *acs2/6* was significantly less than in wt plants. A principal component analysis performed to show the effect of *V. dahliae* on the examined genotypes and based on the relative AUDPC values revealed that *acs2* and *acs2/6* were clustered separately from the *acs6* along the axis describing the 23.8% of the variation (Figure 2). The *acs6* was the most distantly clustered genotype from wt along the PCA axes.

On the other hand, the *acs1* plants were not resistant to *V. dahliae* even if they had less *Verticillium* wilt symptoms than wt until 13 dpi (Figure 1g,h). The mean relative AUDPC value of the *acs1* plants was similar to wt as a result of the enhanced disease severity recorded in the *acs1* after 15 dpi. The principal component analysis revealed that *acs1* was clustered with wt in the axis describing the 31.2% of the variation.

### 2.2. Verticillium dahliae DNA qPCR Quantification

The pathogenicity tests revealed that *acs2*, *acs6*, and *acs2/6* exhibit a partially resistant phenotype to *V. dahliae* infection. To determine whether the reduced disease severity of the *acs* mutants is due to limited fungal growth and colonization in vascular tissues, the plants of the different treatments were harvested at the end of the pathogenicity experiments (21 dpi), and the level of *V. dahliae* DNA was assessed in each genotype by qPCR.

**Figure 3:**
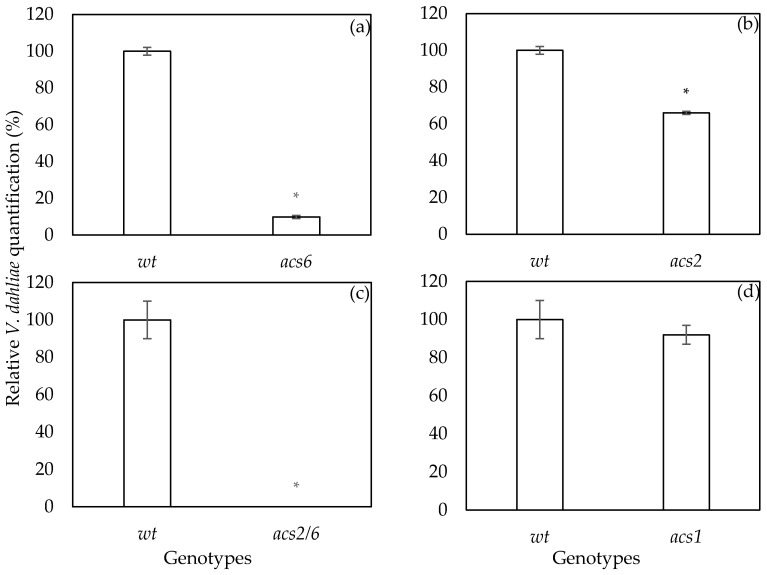
Relative quantification of the *V. dahliae* DNA levels in the Arabidopsis mutants *acs6* (**a**), *acs2* (**b**), *acs2/6* (**c**), *acs1* (**d**), and wild type Col-0 plants. Fungal DNA levels were estimated by qPCR using total DNA isolated from the aerial parts of plants at 21 days post-inoculation. The columns represent the means of 3 biological repeats (with 10 plants per genotype and repeat) and 3 technical repeats per biological repeat (total of nine reactions per treatment). The vertical bars indicate the standard errors. Wild type is set to 100%. Columns with asterisks (*) are significantly different (*p* < 0.05) from wt according to a *t*-test.

*V. dahliae* was present in the vascular tissues of all genotypes; however, in the *acs2*, *acs6*, and *acs2/6* mutants, the levels of the pathogen DNA were significantly lower than those observed in the wt plants (Figure 3). The *acs2/6* had the lowest pathogen levels followed by *acs6*. The *acs2* harbored the highest *V. dahliae* DNA levels among the resistant *acs* mutants; even if the disease severity of *acs2* was as low as it was in the *acs6* and *acs2/6* mutants. On the other hand, the *V. dahliae* DNA levels in the *acs1* mutant were not different from the wt, conferring the susceptibility of the *acs1* to *V. dahliae*.

### 2.3. ACS2 and ACS6 Expression upon Verticillium dahliae Plant Infection

**Figure 4:**
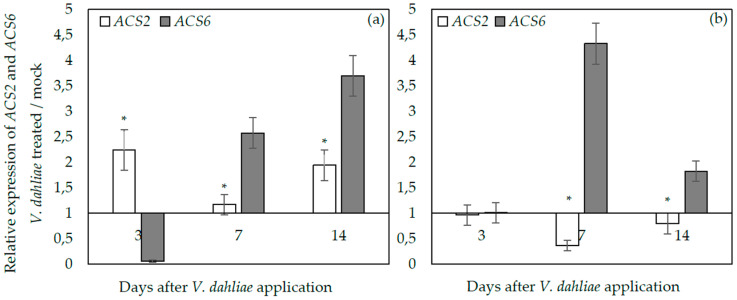
Relative transcript abundance of *ACS2* and *ACS6* in the above ground plant parts (leaves and stems) (**a**) and root (**b**) in wild type *Arabidopsis thaliana* plants inoculated with *Verticillium dahliae*, at 3, 7, and 14 days post-inoculation. Transcript levels of the examined genes were normalized to the expression of the plant gene *At4g26410* measured in the same samples and expressed relative to the normalized transcript levels in mock-treated plants. The experiment was repeated three times with similar results. The columns represent the means of 3 technical repeats of three biological repeats and the vertical bars indicate the standard errors. At each day, columns with asterisks (*) are significantly different (*p* < 0.05) from *ACS6* according to a *t*-test.

To examine whether *ACS2* and *ACS6* are differentially expressed upon *V. dahliae* infection, qPCR analysis was performed on cDNAs prepared from root and above ground (leaves and stems) tissues of Arabidopsis plants (wt) sampled at 3, 7, and 14 dpi. In the above ground tissues, *ACS6* was initially downregulated (3 dpi), while *ACS2* was upregulated two-fold compared to mocks (Figure 4a). The expression levels of *ACS6* increased with time, being 2.5 and 3.7 times higher than mocks, at 7 and 14 dpi, respectively. On the other hand, the expression of *ACS2* decreased at 7 dpi and increased at 14 dpi, 1.9-fold more than mocks.

In the root tissues, the expression of *ACS2* did not follow the same pattern with the above ground tissues, since it was downregulated at 7 and 14 dpi (Figure 4b). On the other hand, the expression of *ACS6* in the *V. dahliae*-treated plants was similar to mocks at 3 dpi and afterwards it was upregulated 4.3-fold and 1.8-fold at 7 and 14 dpi, respectively. Therefore, *ACS6* was upregulated in roots and the above ground tissues at 7 and 14 dpi; while *ACS2* was upregulated in the above ground tissues and downregulated in roots. These observations are further supported by the findings of the ANOVA analysis, showing that *ACS2* expression depends on the type of the tissue and time, while the *ACS6* expression depends on time (Table 1).

### 2.4. Exogenous ACC and AOA Application to wt Arabidopsis thaliana before or after Verticillium dahliae Inoculation

**Figure 5:**
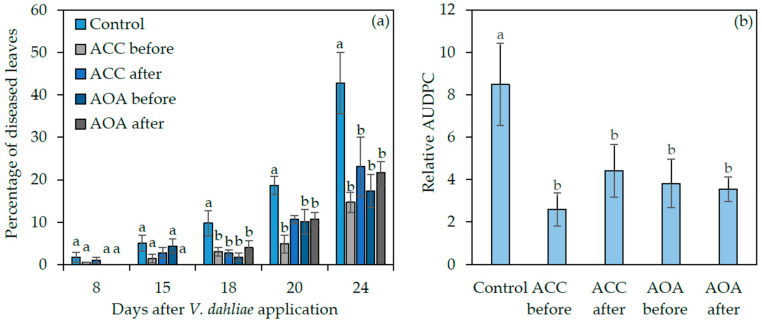
Disease severity (**a**) and relative AUDPC (**b**) of *Arabidopsis thaliana* wild type plants, root-drenched with ACC and AOA before and after inoculation with *Verticillium dahliae*. Vertical bars indicate the standard errors based on twelve replicates. All values were subjected to analysis of variance. At each day in (**a**) and between treatments in (**b**) different letters above columns denote statistically significant differences according to LSD multiple range test at *p* < 0.05.

In order to investigate whether the resistance of *acs2*, *acs6*, and *acs2/6* could be attributed to either low ACC or ethylene production, wt plants were root-drenched with ACC or aminooxyacetic acid (AOA), the competitive inhibitor of ACS [15], before or after *V. dahliae* infection. The first *Verticillium* wilt symptoms appeared in the control and ACC-treated plants at 8 dpi, while the AOA-treated plants were asymptomatic at that time point (Figure 5a). Overall, the disease severity progressed slower in ACC and AOA treatments compared to controls. At 24 dpi, the percentage of disease severity was 43% in the control treatment; while in the treatments where AOA and ACC were applied before pathogen inoculation, the disease severity was 23% and 15%, respectively. Similarly, the application of AOA and ACC after *V. dahliae* application resulted in 2.5- and 2-fold, respectively, lower symptoms compared to control at 24 dpi (Figure 5a).

The relative AUDPC that was adopted to comprehensively evaluate the disease severity, confirmed the previous observations. The mean relative AUDPC value for the plants treated with ACC before or after *V. dahliae* inoculation was 3.3- and 1.9-fold, respectively, lower than control; the respective values in the AOA-treated plants were 2.2- and 2.4-fold lower than control (Figure 5b).

## 3. Discussion

The role of *ACS* in plant–pathogen interactions has been previously discussed upon plant infection by *B. cinerea* and *P. syringae* pv *tomato* [12,13]. It has been shown that *ACS2* and *ACS6* positively regulate defense responses against both pathogens. On the contrary, our results suggest a negative role for *ACS2* and *ACS6* in plant disease resistance against *V. dahliae*.

In our experiments, we initially screened the resistance level of *acs2*, *acs6*, *acs2/6*, and *acs1* against *V. dahliae*. The *acs2*, *acs6*, and *acs2/6* showed significantly less symptoms than wt. Therefore, the pathogenicity experiments suggest a partial resistance genotype for *acs2* and *acs6*. This is an interesting observation since *ACS2* and *ACS6* are components of the signal transduction pathway leading to disease defense responses [14]. In the defense signaling cascade, *ACS2* and *ACS6* interact with the mitogen activated protein kinases (MPK) 3 (MPK3) and 6 (MPK6) [14]. It is known that MPK3 and MPK6 positively regulate ethylene production through the phosphorylation-mediated stabilization of *ACS2* and *ACS6* [14,16]. Similar to *acs2* and *acs6*, the Arabidopsis mutants *mpk3* and *mpk6* are resistant to *V. dahliae* [17]. Therefore, the pathogen may use the interaction of *ACS2* and *ACS6* with MPK3 and MPK6 to promote disease.

In contrast to the resistant phenotype of *acs2* and *acs6*, the *acs1* mutant was susceptible to *Verticillium*; even if it showed less symptoms than wt at the early disease recordings. It is not surprising that *acs1* is susceptible to *Verticillium*, whereas the *acs2* and *acs6* are resistant; since the *ACS1* is enzymatically inactive due to the absence of a highly conserved tripeptide Thr-Asn-Pro (TNP) [18] and it is not phosphorylated by MPK3 and MPK6 as it happens with *ACS2* and *ACS6*.

The pathogenicity experiments suggest that *ACS2* and *ACS6* influence the outcome of *V. dahliae* infection. However, the observed reduction of disease symptoms in the *acs2* and *acs6* plants compared to wt could be the outcome of a tolerance phenomenon rather than a resistance response. The plant tolerance to *V. dahliae* has been previously addressed in Arabidopsis when the disease response of different ecotypes to *V. dahliae* was studied [19]. In this study, the ecotype with the fewer wilt symptoms among the examined ecotypes was harboring endophyticaly as much *Verticillium* as the most susceptible ecotype [19]. For this purpose, we quantified the endophytic presence of *V. dahliae* in *acs2*, *acs6*, *acs2/6*, *acs1*, and wt plants at the end of the experiment. The qPCR analysis revealed lower levels of *V. dahliae* DNA in *acs2*, *acs6*, and *acs2/6* plants compared to wt. These results suggest that the observed reduction in *Verticillium* wilt symptoms in the *acs* mutants was due to partial resistance, as it has been also described in the case of *mpk3*, *mpk6*, *etr1*, and *efr1* [5,17]. The phenomenon of partial increase in resistance is linked to quantitative disease resistance (QDR), reported in several studies on Arabidopsis pathosystems including *P. syringae*, *Erisyphe cichoracearum*, and *B. cinerea* [20,21,22].

Ethylene production by plants, at the early stages of plant–pathogen interaction, has been associated with the induction of defense mechanisms, while its production at the later stages of the infection process may act in favor of the pathogen [23]. In our experiments, the upregulation of *ACS6* and *ACS2* in the examined tissues after pathogen infection was observed, indicating that higher ACC or ethylene production after *V. dahliae* infection could enhance disease. Since *ACS2* and *ACS6* are involved in ethylene biosynthesis, we investigated whether exogenous application of AOA, an ACC inhibitor, could mimic the results obtained from the pathogenicity experiments with *acs2* and *acs6* mutants. Indeed, our results showed that application of AOA after or before *V. dahliae* infection reduces disease severity compared to the untreated controls. However, the application of ACC to plants, instead of reversing the phenotype observed upon AOA treatment, resulted in significant reduction of disease severity. Likewise, Robison et al. (2001) [24] suggested that long-term inhibition of ethylene production through AVG (aminoethoxyvinylglycine) treatment, an ACC inhibitor, combined with a transient burst of ethylene at the time of infection upon ACC application, could reduce *Verticillium* wilt disease symptoms in tomato plants. A more recent study suggested that ACC produced by *V. dahliae* can act as a negative regulator of virulence [25]. In the same study, the authors postulated that *V. dahliae* strains overexpressing ACC deaminase might promote disease by reducing plant ACC levels in the roots [25]. Consequently, our results suggest that the mechanism underpinning the partial resistance of *acs2* and *acs6* is likely ethylene depended rather than ACC related, since ACC application led to decreased disease severity.

*ACS2* and *ACS6* can be integrated in an earlier proposed virulence model of *V. dahliae*, suggesting that the elongation factor Tu (EF-Tu) receptor (EFR), MPK3, and MPK6 are susceptibility factors for *V. dahliae* [16]. The EFR binds the bacterial protein EF-Tu [26], it is also known that ET modulates EFR-triggered immunity [27]. Interestingly, a gene (VDAG_01458.1) homologous to EF-Tu that can be detected by EFR and trigger a virulence cascade in the plant, including MPK3 and MPK6, exists in the *V. dahliae* genome. Arabidopsis MAP kinases MPK3 and MPK6 are activated within 5 min of elf18 treatment [28]. Therefore, the initial binding of the putative *V. dahliae* EF-Tu to EFR can lead to the activation of MPK3 and MPK6 that in turn phosphorylates the ACS proteins, resulting in ET biosynthesis [29,30]. Among the receptors of ET in plant cells is ETR1, a susceptibility factor for *V. dahliae* and *V. longisporum* [5,6]. As proposed earlier, ET synthesis and perception might have a significant role in *Verticillium* pathogenesis and plant resistance or tolerance [29]. The use of Arabidopsis mutants for each step of this signaling cascade showed their resistance against *V. dahliae*; therefore, *EFR*, *MPK3*, and *MPK6*, along with *ACS2* and *ACS6*, might be manipulated by *V. dahliae* to promote disease.

In conclusion, our study is in agreement with an increasing number of studies showing that ACC possess a signaling role in plant defense beyond its function in ethylene biosynthesis. However, the partial resistance of *acs2* and *acs6* suggests a negative regulatory role for *ACS2* and *ACS6* in plant defense against *V. dahliae,* which seems to be ethylene depended. This is the first published study proposing that genes involved in ethylene biosynthesis and not ethylene perception contribute to *V. dahliae* disease development; further studies are needed to unravel the plant defense mechanisms that are activated in *acs2* and *acs6* mutants upon *V. dahliae* infection.

## 4. Materials and Methods

### 4.1. Fungal Culture

A *V. dahliae* strain isolated from *Raphanus sativus* L. was used in the experiments [30]. The fungal strain was stored at −80 °C as a spore suspension in 25% aqueous glycerol [31]. For the experiments, the fungus was transferred to potato dextrose agar (PDA) (Merck, Darmstadt, Germany) at 24 °C for 5 days and subsequently a suspension of 10^7^ conidia/mL of distilled sterile water was prepared from a culture grown for 5 days at 24 °C in a sucrose sodium nitrate liquid medium [32].

### 4.2. Seeds Origin and Plant Growth Conditions

*A. thaliana* Col-0, wt, and the *acs2*, *acs6, acs2/6* and *acs1* mutants [10] were obtained from the Nottingham Arabidopsis Stock Centre, Nottingham, UK. The stock numbers are CS16564 (*acs2*), CS16569 (*acs6*), CS16581 (*acs2/6*) and CS16563 (*acs1*). All seeds were stored at 4 °C. For the bioassays, *A. thaliana* seeds were sown in pots (9 × 9 × 10 cm) containing pasteurized soil mix of humus and perlite (3:1) and were maintained at 22 °C with a 16-h photoperiod at 60–70% relative humidity in a controlled-environment growth chamber. After 10 days, the plants were singled to plastic pots with approximately 80 cm^3^ of pasteurized soil mix of humus and perlite (3:1).

For ACC and AOA experiments, Arabidopsis Col-0 seeds were sown directly into 9 cm-diameter pots (Teku, VCH 9 pots), containing approximately 330 mL soil (Plantaflor Potting Soil, Germany) per pot. After 4 days of stratification at 4 °C, the pots were placed in a growth room set at 22 °C, 65–70% RH, and a 16-h photoperiod with photon flux density of 100 ± 20 μmol m^−2^ s^−1^. Plants were watered every two days to maintain 70% of soil humidity.

### 4.3. Pathogenicity Experiments

Twenty-day-old plants were root-drenched either with 10 mL of 10^7^ *V. dahliae* conidia mL^−1^ sterile distilled water per plant or sterile distilled water (mocks) [5]. The experiment was repeated three times with 10 plants per genotype (wt, *acs2*, *acs2*, *acs2/6*, *acs1*) and treatment (*V. dahliae*; mocks) per replication (a total of 30 plants treated with *V. dahliae* and 30 mock inoculated plants per genotype). The monitoring of *Verticillium* wilt symptoms started at 7 dpi until 21 dpi. Disease severity at each observation was calculated from the number of leaves that showed *Verticillium* symptoms as a percentage of the total number of leaves of each plant. Subsequently, the area under the disease progress curve (AUDPC) was calculated by the trapezoidal integration method [33] for each plant. Disease was expressed as a percentage of the maximum possible area for the whole period of the experiment, which is referred to as the relative AUDPC [17].

The effect of ACC and AOA on disease development was evaluated by applying a solution of 100 μM ACC or AOA by root drenching (10 mL per plant) two-week-old plants 24 h prior or 24 h after fungal inoculation. Pathogen inoculation and disease assessment were performed as it is described above. Six plants per treatment were used, and the experiment was repeated twice.

### 4.4. DNA Extraction and qPCR Fungal Quantification

The above ground plant parts of 10 plants per genotype were harvested at 21 dpi (end of the pathogenicity experiments) and pooled to one sample for qPCR quantification of the *V. dahliae* DNA endophytic levels. In brief, the plants were cut at soil level, rinsed with sterile distilled water, and ground to a fine powder, using an autoclaved mortar and pestle in the presence of liquid nitrogen.

Total DNA was isolated according to Dellaporta et al. (1983) [34] and the concentration was estimated by using NanoDrop UV spectrophotometry. qPCR assays for the quantification of *V. dahliae* were conducted as described previously [17], using the pair of primers ITS1-F 5′–AAAGTTTTAATGGTTCGCTAAGA–3′ and ST-VE1-R 5′–CTTGGTCATTTAGAGGAAGTAA–3′ designed for the ITS1 and ITS2 regions of the 5.8S ribosomal RNA gene (Z29511) of *V. dahliae*. qPCRs were performed in an Applied Biosystems StepOnePlus thermocycler and for the amplification reactions, FastGene IC Green qPCR universal mix (NIPPON Genetics EUROPEGmbH) was used. The results were analyzed with the StepOne v.2.3 qPCR software. For sample calibration, the Arabidopsis gene *At4g26410*, previously described as a stable reference gene [35], was detected using the primer pair 5′-GAGCTGAAGTGGCTTCCATGAC-3′ and 5′-GGTCCGACATACCCATGATCC-3′. PCR cycling started with an initial step of denaturation at 95 °C for 2 min, followed by 40 cycles of 95 °C for 5 s and 60 °C for 30 s. PCR efficiency for each amplicon was calculated by employing the linear regression method on log (fluorescence) per cycle number data, using the Lin-Reg PCR software [36]. Three biological repeats were conducted with 10 plants per genotype and repeat (a total of 30 plants per genotype) and three technical repeats per biological repeat. The absence of nonspecific products and primer dimers was confirmed by the analysis of melting curves. The relative *V. dahliae* DNA quantity, calculated by using the formula 2^−ΔCt^, was expressed as a percentage compared to wt; while the value (2^−ΔCt^) of the wt was set to 100% [17].

### 4.5. Determination of Transcript Levels Using RT-PCR Assay

Ten *A. thaliana* wt plants from each treatment (*V. dahliae* and mock) and experimental replication (a total of 3 replications) were harvested for RNA analysis and pooled to one sample at 3, 7, and 14 dpi. For each sampled plant, the above-ground parts (stem and leaves) were cut at soil level, and roots (main and secondary) were shortly rinsed with sterile distilled water to remove soil particles, immediately frozen in liquid nitrogen, and stored at −80 °C. Subsequently, the pooled tissues were ground with liquid nitrogen and total RNA was extracted from 100 mg of the homogenized tissues using TRIzol^®^ Reagent (Invitrogen, Paisley, Renfrewshire, UK), according to the manufacturer’s instructions.

The RNA samples were treated with DNase I (Invitrogen) to eliminate traces of contaminating genomic DNA. The RNA concentration was measured on a Nanodrop ND-1000 spectrophotometer (Saveen Werner, Malmö, Sweden). First-strand cDNA was synthesized using SuperScript II (Invitrogen) following the manufacturer’s procedure. The primers used for real-time PCR were *ACS2* (*At1g01480*; 5-GGATGGTTTAGGATTTGCTTTG-3 and 5-GCACTCTTGTTCTGGATTACCTG-3), and *ACS6* (*At4g11280*; 5-GTTCCAACCCCTTATTATCC-3 and 5-CCGTAATCTTGAACCCATTA-3) [12]. PCR cycling started with an initial step of denaturation at 95 °C for 2 min, followed by 40 cycles of 95 °C for 5 s and 60 °C for 30 s. PCR efficiency for each amplicon was calculated by employing the linear regression method on log (fluorescence) per cycle number data, using the Lin-Reg PCR software [36]. Three biological repeats were conducted with 10 plants per sampling time point (3, 7, 14 dpi), treatment (*V. dahliae* and mock inoculated plants), and repeat (a total of 30 plants per treatment per sampling time point). Three technical repeats were conducted per biological repeat. The absence of nonspecific products and primer dimers was confirmed by the analysis of melting curves. The relative *ACS2* and *ACS6* expression levels (2^−ΔCt^) in the *V. dahliae*-treated plants were expressed relative to the normalized transcript levels of *ACS2* and *ACS6* in the mock-inoculated plants.

### 4.6. Statistics

Data on disease severity, relative AUDPC, *V. dahliae* qPCR quantification, and relative gene expression of *ACS2* and *ACS6* were analyzed with a two-sample *t*-test (*p* < 0.05); except of the relative AUDPC in the AOA/ACC experiment, where the values of the different treatments were subjected to analysis of variance followed by LSD multiple range test (*p* < 0.05). A standardized PCA was performed on the relative AUDPC values of the examined genotypes (wt, *acs1*, *acs2*, *acs6*, *acs2/6*) using the XLSTAT software.

## Figures and Tables

**Table 1:** Analysis of variance for gene expression of *ACS2* and *ACS6* in the examined tissues (above ground plant tissues and roots) in the three examined time points (3, 7, and 14 days post-inoculation (dpi)). The analysis was based on the relative gene expression results (2^−ΔCt^) of 3 technical repeats of three biological repeats.

	*ACS2*		*ACS6*	
Source	df	*F*	df	*F*
Tissue	1	413.06 **	1	2.53
Time	2	90.09 **	2	118.14 **
Tissue × Time	2	7.29 *	2	46.80 **
Error	48		48	
Total	54		54	

The asterisks (*) and (**) denote significance at *p* < 0.01, and 0.001 levels, respectively, according to the F test; df: degrees of freedom; *F*: variance of the group means / mean of the within group variances.
